# Supplementary material for: Quality Evaluation of Hainan Robusta Coffee Bean Oil Produced by Ultrasound Coupled with Coconut Oil Extraction
Source: Foods. 2023 Jun 1;12(11):2235. doi: 10.3390/foods12112235 (PMC10253148; doi:10.3390/foods12112235)
Supplement: Supplementary file 1 [file foods-12-02235-s001.zip › foods-2399456-supplementary.pdf]

Supplement material

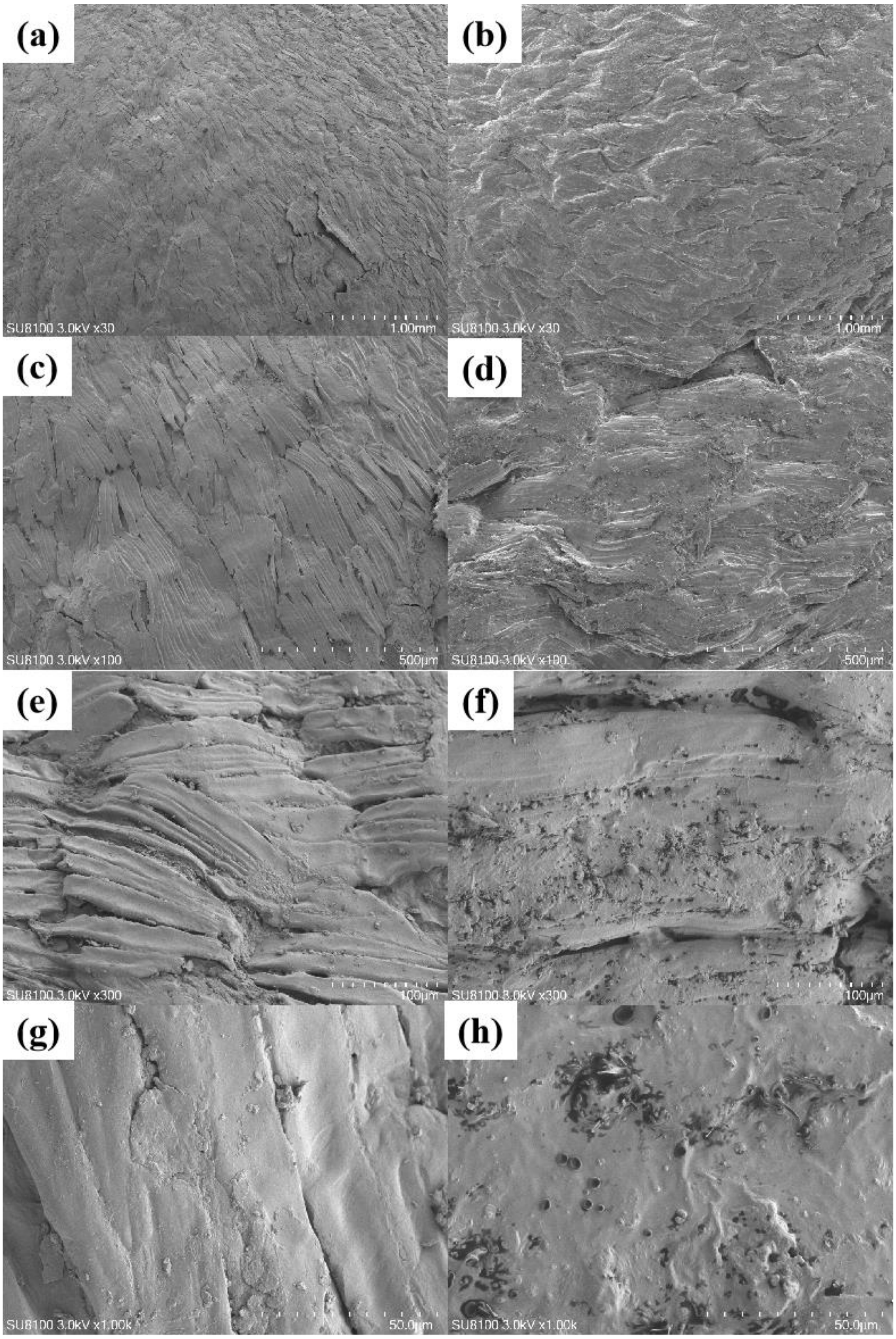

Figure S1: Changes of coffee bean microstructure before and after ultrasound treatment. coffee bean microstructure before ultrasound treatment (a)(c)(e)(g); coffee bean microstructure after ultrasound treatment(90min) (b)(d)(f)(h)
